# Development and validation of a nomogram for predicting diabetic foot ulcer risk in patients with type 2 diabetes mellitus

**DOI:** 10.3389/fendo.2025.1555163

**Published:** 2025-06-30

**Authors:** Xiuli Feng, Renhao Zhao, Teng Yang, Na Wang, Guofeng Wang

**Affiliations:** ^1^ Department of Endocrinology, Lianyungang Clinical College of Nanjing Medical University/The First People’s Hospital of Lianyungang, Lianyungang, China; ^2^ Department of Endocrinology, The First People’s Hospital of Yancheng, Yancheng, China; ^3^ Department of Endocrinology, Postgraduate Training Base of Lianyungang First People’s Hospital of Jinzhou Medical University, Lianyungang, China

**Keywords:** type 2 diabetes mellitus, diabetic foot ulcer, nomogram, risk factors, prediction

## Abstract

**Objective:**

To identify the risk factors of diabetic foot ulcer (DFU) in patients with type 2 diabetes mellitus (T2DM) and to develop and validate a nomogram prediction model for DFU occurrence in primary care setting.

**Methods:**

We conducted a single-center retrospective study enrolling 547 T2DM patients hospitalized at The First People’s Hospital of Lianyungang from January 2019 to April 2022. Patients were randomly divided (3:1) into modeling (n = 411) and validation (n = 136) cohorts, and further stratified by DFU status. Thirty-four clinical variables were extracted for analysis. LASSO regression with tenfold cross-validation identified key features, followed by multivariate logistic regression to determine independent DFU risk factors. A nomogram model was developed using R software, and its performance was evaluated using the area under the receiver operating characteristic curve (AUC), calibration curves, the Hosmer–Lemeshow goodness-of-fit test, and decision curve analysis (DCA).

**Results:**

Among 547 T2DM patients, 150 (27.4%) developed DFU. Multivariate analysis identified seven independent risk factors: age (odds ratio [OR] = 1.032, 95% confidence interval [CI]: 1.005–1.062, *P* = 0.021), white blood cell (WBC) (OR = 1.127, 95% CI: 1.006–1.270, *P* = 0.043), ankle-brachial index (ABI) (OR = 5.447, 95% CI: 2.186–14.340, *P* < 0.001), urine albumin-to-creatinine ratio (UACR) (OR = 2.049, 95% CI: 1.062–3.936, *P* = 0.031), family history of diabetes (OR = 3.405, 95% CI: 1.666–7.039, *P* < 0.001), diabetic peripheral neuropathy (DPN) (OR = 5.084, 95% CI: 2.673–9.805, *P* < 0.001), and albumin (ALB) (OR = 0.850, 95% CI: 0.786–0.915, *P* < 0.001). The developed nomogram demonstrated excellent discrimination (AUC = 0.917 and 0.956 for modeling and validation cohorts). Internal validation confirmed good model reliability (C-index = 0.917). Calibration curves showed strong agreement between predicted and observed outcomes (Hosmer–Lemeshow *P* = 0.649 and 0.345). DCA indicated a consistently higher net benefit across threshold probabilities of 0 to 0.8, underscoring the model’s potential clinical utility.

**Conclusions:**

The nomogram prediction model developed in this study demonstrates excellent performance and strong clinical applicability. It provides an effective tool to identify high-risk T2DM patients for DFU and guide early preventive interventions.

## 1 Introduction

Diabetic foot ulcer (DFU), a severe complication of diabetes mellitus, characterized by deep tissue damage resulting from peripheral arterial disease (PAD) and lower-extremity neuropathy. It is associated with high rates of morbidity, disability, and mortality, imposing a substantial medical and economic burden (1–4). The lifetime risk of DFU in diabetic patients is estimated to range from 19% to 34%. Approximately half of these cases progress to infection, requiring hospitalization, and the recurrence rate reaches up to 65% within five years (1, 5). DFU is responsible for approximately 85% of non-traumatic lower-extremity amputations (1). The five-year post-amputation mortality rate ranges from 39% to 80%, exceeding that of most malignancies (5, 6). In China, the annual incidence of DFU is 8.1%, increasing to 25% among the elderly. The one-year recurrence, amputation, and mortality rates stand at 31.6%, 5.1%, and 14.4%, respectively (7). According to the International Diabetes Federation’s 2019 report, global diabetes-related healthcare expenditures reached USD 760 billion, with DFU accounting for 30%–40% of this total (8).

Given the substantial burden associated with DFU, early identification of high-risk individuals and timely intervention are critical for effective clinical prevention and management. Evidence suggests that more than 50% of DFU cases and related amputations could be prevented through effective risk assessment and management (9). Studies have shown that routinely collected clinical data have strong predictive value for both the occurrence and progression of DFU, providing a valuable opportunity for accurate risk stratification (10). To this end, a variety of predictive models have been developed, including the risk stratification score by Boyko et al. (11), the logistic regression model by Crawford et al. (12), the weighted scoring system by Shi et al. (13), and the nomogram developed by Jiang et al. (14). Despite their potential clinical value, the applicability of these tools is limited. Specifically, many models rely on data from Western populations or tertiary hospitals in large cities, limiting their generalizability to smaller cities or primary healthcare settings. Moreover, these models often depend on dichotomous variables or subjective scoring, neglecting continuous variables. This reliance may compromise both predictive accuracy and clinical usability.

Multiple studies have consistently demonstrated that poor glycemic control, longer duration of diabetes, PAD, and diabetic peripheral neuropathy (DPN) are major risk factors for DFU (15–17). Studies by Lin et al. (18) and Shao et al. (19) have further validated the broad applicability of these factors across various Chinese subpopulations. However, with the ongoing shifts in patient demographics and increasing unequal access to healthcare services across regions, current predictive models reveal significant limitations, such as narrow variable selection, lack of external validation, and insufficient longitudinal or prospective study designs. Furthermore, this gap is most evident among hospitalized patients in small- and medium-sized cities, where locally validated DFU risk prediction tools are scarce, hindering the implementation of individualized management and early intervention strategies in clinical practice.

Therefore, this study aimed to develop a concise and clinically applicable nomogram using routinely collected clinical variables from hospitalized type 2 diabetes mellitus (T2DM) patients in a tertiary hospital in Lianyungang, China. The model was designed to facilitate early DFU risk identification and provide a robust framework for individualized prevention strategies in resource-limited healthcare settings, particularly small- and medium-sized urban hospitals.

## 2 Materials and methods

### 2.1 Study design and participants

We conducted a single-center retrospective study at The First People’s Hospital of Lianyungang, enrolling consecutive T2DM patients hospitalized between January 2019 to April 2022. Patients were randomly assigned in a 3:1 ratio to a modeling cohort (n = 411, 75%) and a validation cohort (n = 136, 25%) using simple randomization. Each cohort was further stratified into DFU and non-DFU groups based on DFU diagnosis.

The inclusion criteria were as follows: (1) Age ≥ 18 years; (2) Diagnosis of T2DM according to the 1999 World Health Organization (WHO) criteria; (3) Diagnosis of DFU based on the Chinese Guidelines for the Prevention and Treatment of Type 2 Diabetes; (4) Complete clinical data, including demographic and clinical characteristics, diabetic complications, physical examinations, laboratory parameters and treatment modalities.

The exclusion criteria were as follows: (1) Presence of acute diabetic complications, such as diabetic ketoacidosis or hyperosmolar hyperglycemic state; (2) Presence of severe infections (including active infection or sepsis), gangrenous changes in the lower extremities, or a history of lower limb amputation; (3) Presence of malignancy, end-stage renal disease, advanced heart failure, advanced liver disease, or other severe systemic conditions potentially influencing DFU risk assessment; (4) Pregnancy or lactation; (5) Incomplete clinical data or absence of key variables required for analysis.

### 2.2 Data collection

The dependent variable was defined as the occurrence of DFU. Thirty-four independent variables were retrospectively extracted from the patients’ electronic medical records. These variables included demographic and clinical characteristics (age, gender, diabetes duration, hypertension, coronary artery disease [CAD], cerebral infarction [CI], family history of diabetes, smoking, alcoholism), diabetic complications (DPN, diabetic retinopathy [DR]), physical examinations (body mass index [BMI], ankle-brachial index [ABI]), laboratory parameters (fasting plasma glucose [FPG], fasting C-peptide [FCP], glycated hemoglobin [HbA1c], hemoglobin [Hb], red blood cell [RBC], white blood cell [WBC], platelet [PLT], alanine aminotransferase [ALT], aspartate aminotransferase [AST], alkaline phosphatase [ALP], albumin [ALB], blood urea nitrogen [BUN], serum creatinine [Scr], uric acid [UA], total cholesterol [TC], triglyceride [TG], high-density lipoprotein cholesterol [HDL-C], low-density lipoprotein cholesterol [LDL-C], urine albumin-to-creatinine ratio [UACR]), and treatment modalities (oral hypoglycemic agent [OHA] use, insulin [INS] use).

All data were systematically extracted into Microsoft Excel files and independently verified by two researchers to ensure accuracy.

### 2.3 Sample size justification

The sample size was determined based on the requirements of multivariate logistic regression and nomogram development, following the established guideline by Peduzzi et al. (1996) (20). Accordingly, the minimum sample size for logistic regression was calculated using the following formula:


$$\text{N }=\;\frac{10\;\times \text{ k}}{\text{p}}$$

where N represents the required sample size, k represents the number of independent variables included in the model, and p indicates the proportion of positive outcomes (i.e., the DFU event rate observed in our study population).

Given that 34 candidate variables were considered and the observed DFU incidence reached 27.4%, we calculated the minimum sample size as follows:


$$\text{N}=\frac{10\;\times \;34}{0.274}\approx 1241$$

To address model optimization and mitigate overfitting risk, we employed LASSO regression method for variable selection. This process significantly refined the final multivariate model to include only eight predictors. Accordingly, the adjusted minimum required sample size was:


$$\text{N }=\;\frac{10\;\times \;8}{0.274}\;\approx \;292$$

Our final sample size of 547 patients exceeded this minimum requirement, ensuring the adequacy of the sample for robust multivariate analysis and reliable nomogram development.

### 2.4 Statistical analysis

All statistical analyses were performed using SPSS version 25.0 (IBM Corp., Armonk, NY, USA) and R version 4.1.3 (R Foundation for Statistical Computing, Vienna, Austria). Categorical variables were compared using either chi-square test or Fisher’s exact test, as appropriate. Continuous variables were compared using the Student’s t-test or the Mann-Whitney U test, depending on the normality of the data distribution. To optimize variable selection and mitigate overfitting, LASSO regression with tenfold cross-validation was used to identify optimal predictors of DFU. These selected predictors were subsequently incorporated into a multivariable logistic regression model to identify independent risk factors for DFU. Based on this final model, a nomogram was constructed for DFU risk prediction. The predictive performance of the nomogram was evaluated using the area under the receiver operating characteristic (ROC) curve (AUC). Internal validation was conducted using the bootstrap method with 1000 resamples, and the concordance index (C-index) was calculated to assess model discrimination. A calibration curve was plotted to evaluate the agreement between predicted and observed probabilities. The Hosmer–Lemeshow goodness-of-fit test was performed to assess the model’s calibration accuracy. Decision curve analysis (DCA) was used to evaluate the clinical utility of the nomogram. A two-sided *P* value < 0.05 was considered statistically significant.

## 3 Results

### 3.1 Baseline characteristics

A total of 547 patients with T2DM were enrolled in this study, with 411 assigned to the modeling cohort and 136 to the validation cohort. Participants in each cohort were stratified into DFU and a non-DFU groups based on DFU diagnosis. Specifically, in the modeling cohort, 110 patients had DFU and 301 did not, while in the validation cohort, 37 patients had DFU and 99 did not. No significant difference in DFU proportion was observed between the two cohorts (*P* > 0.05). Furthermore, baseline characteristics—demographic and clinical characteristics, diabetic complications, physical examinations, laboratory parameters and treatment modalities—showed no statistically significant differences between the two cohorts (*P* > 0.05; **Table 1**). This comparability between cohorts established a robust basis for model development and validation.

**Table 1:** Baseline characteristics of the modeling and validation cohorts.

Variable	Modeling cohort (n = 411)	Validation cohort (n = 136)	*P* value
DFU, n (%)			1.000
Yes	113 (27.5)	37 (27.2)	
No	298 (72.5)	99 (72.8)	
Gender, n (%)			0.471
Male	261 (63.5)	81 (59.6)	
Female	150 (36.5)	55 (40.4)	
Hypertension, n (%)			0.862
Yes	205 (49.9)	66 (48.5)	
No	206 (50.1)	70 (51.5)	
CAD, n (%)			0.767
Yes	45 (10.9)	13 (9.6)	
No	366 (89.1)	123 (90.4)	
CI, n (%)			0.978
Yes	49 (11.9)	17 (12.5)	
No	362 (88.1)	119 (87.5)	
ABI, n (%)			0.850
≥1.3 or ≤0.9	56 (13.6)	17 (12.5)	
0.9–1.3	355 (86.4)	119 (87.5)	
Smoking, n (%)			0.312
Yes	117 (28.5)	32 (23.5)	
No	294 (71.5)	104 (76.5)	
Alcoholism, n (%)			0.468
Yes	144 (35.0)	53 (39.0)	
No	267 (65.0)	83 (61.0)	
UACR, n (%)			0.612
≥30mg/g	154 (37.5)	47 (34.6)	
<30mg/g	257 (62.5)	89 (65.4)	
Family history of diabetes, n (%)			0.489
Yes	89 (21.7)	25 (18.4)	
No	322 (78.3)	111 (81.6)	
DR, n (%)			0.458
Yes	112 (27.3)	32 (23.5)	
No	299 (72.7)	104 (76.5)	
DPN, n (%)			0.333
Yes	132 (32.1)	37 (27.2)	
No	279 (67.9)	99 (72.8)	
OHA use, n (%)			0.551
Yes	283 (68.9)	98 (72.1)	
No	128 (31.1)	38 (27.9)	
INS use, n (%)			0.679
Yes	165 (40.1)	58 (42.6)	
No	246 (59.9)	78 (57.4)	
Age (years)	56.66 ± 13.51	57.57 ± 13.6	0.695
BMI (kg/m²)	25.97 ± 3.85	25.77 ± 3.96	0.658
Diabetes duration (months)	119.55 ± 107.75	129.89 ± 96.98	0.144
FPG (mmol/L)	10.01 ± 4.26	9.73 ± 4.02	0.418
FCP (pmol/L)	831.11 ± 624.31	755.96 ± 663.36	0.067
HbA1c (%)	9.44 ± 2.28	9.74 ± 2.36	0.154
Hb (g/L)	132.86 ± 18.98	129.74 ± 19.15	0.167
RBC (×10^9^/L)	4.52 ± 0.65	4.44 ± 0.64	0.211
WBC (×10^9^/L)	7.16 ± 2.74	7.04 ± 2.69	0.345
PLT (×10^9^/L)	225.7 ± 72.72	224.26 ± 77.08	0.870
ALT (U/L)	26.02 ± 44.18	22.77 ± 24.53	0.388
AST (U/L)	22.04 ± 30.92	19.52 ± 11.08	0.559
ALP (U/L)	88.91 ± 43.7	87.73 ± 30.29	0.634
ALB (g/L)	38.52 ± 5.22	38.88 ± 9.58	0.793
BUN (mmol/L)	6.62 ± 3.01	6.01 ± 1.9	0.074
Scr (μmol/L)	66.53 ± 48.89	95.85 ± 373.8	0.422
UA (μmol/L)	307.08 ± 102.56	313.92 ± 194.89	0.513
TG (mmol/L)	2.09 ± 2.04	1.92 ± 1.72	0.273
TC (mmol/L)	4.41 ± 1.2	4.41 ± 1.22	0.942
HDL-C (mmol/L)	1.03 ± 0.28	0.99 ± 0.22	0.502
LDL-C (mmol/L)	2.51 ± 0.78	2.51 ± 0.79	0.905

### 3.2 Univariate analysis and LASSO regression in the modeling cohort

Within the modeling cohort, patients with DFU showed higher proportions of males, older age, and longer diabetes duration compared to non-DFU patients. They also exhibited elevated levels of UACR, Scr, BUN, WBC, and PLT, along with increased prevalence of abnormal ABI, Hypertension, CAD, CI, DR, DPN, family history of diabetes, and INS use. Conversely, significantly lower levels of BMI, Hb, RBC, ALB, ALT, AST, and TG were observed in DFU patients (*P* < 0.05; **Table 2**).

**Table 2:** Baseline characteristics of patients in the DFU and non-DFU groups (modeling cohort).

Variable	DFU group (n=113)	Non-DFU group (n=298)	*P* value
Gender, n (%)			0.045
Male	81 (71.7)	180 (60.4)	
Female	32 (28.3)	118 (39.6)	
Hypertension, n (%)			<0.001
Yes	74 (65.5)	131 (44.0)	
No	39 (34.5)	167 (56.0)	
CAD, n (%)			0.012
Yes	20 (17.7)	25 (8.4)	
No	93 (82.3)	273 (91.6)	
CI, n (%)			<0.001
Yes	26 (23.0)	23 (7.7)	
No	87 (77.0)	275 (92.3)	
ABI, n (%)			<0.001
≥1.3 or ≤0.9	44 (38.9)	12 (4.0)	
0.9–1.3	69 (61.1)	286 (96.0)	
Smoking, n (%)			0.568
Yes	35 (31.0)	82 (27.5)	
No	78 (69.0)	216 (72.5)	
Alcoholism, n (%)			0.628
Yes	37 (32.7)	107 (35.9)	
No	76 (67.3)	191 (64.1)	
UACR, n (%)			<0.001
≥30 mg/g	75 (66.4)	79 (26.5)	
<30 mg/g	38 (33.6)	219 (73.5)	
Family history of diabetes, n (%)			<0.001
Yes	45 (39.8)	44 (14.8)	
No	68 (60.2)	254 (85.2)	
DR, n (%)			<0.001
Yes	46 (40.7)	66 (22.1)	
No	67 (59.3)	232 (77.9)	
DPN, n (%)			<0.001
Yes	82 (72.6)	50 (16.8)	
No	31 (27.4)	248 (83.2)	
OHA use, n (%)			0.174
Yes	84 (74.3)	199 (66.8)	
No	29 (25.7)	99 (33.2)	
INS use, n (%)			0.001
Yes	61 (54.0)	104 (34.9)	
No	52 (46.0)	194 (65.1)	
Age (years)	62.02 ± 11.40	54.63 ± 13.71	<0.001
BMI (kg/m²)	24.98 ± 3.70	26.34 ± 3.85	0.001
Diabetes duration (months)	168.94 ± 111.28	100.83 ± 100.39	<0.001
FPG (mmol/L)	9.70 ± 4.62	10.13 ± 4.11	0.359
FCP (pmol/L)	745.78 ± 634.10	863.47 ± 618.55	0.088
HbA1c (%)	9.54 ± 2.61	9.40 ± 2.14	0.582
Hb (g/L)	123.74 ± 19.53	136.32 ± 17.61	<0.001
RBC (×10^9^/L)	4.17 ± 0.68	4.65 ± 0.59	<0.001
WBC (×10^9^/L)	8.54 ± 3.61	6.63 ± 2.11	<0.001
PLT (×10^9^/L)	246.54 ± 90.13	217.80 ± 63.32	<0.001
ALT (U/L)	16.27 ± 10.84	29.71 ± 51.00	0.006
AST (U/L)	17.15 ± 8.90	23.89 ± 35.74	0.048
ALP (U/L)	92.15 ± 34.34	87.68 ± 46.76	0.355
ALB (g/L)	34.05 ± 4.86	40.22 ± 4.27	<0.001
BUN (mmol/L)	7.70 ± 3.69	6.22 ± 2.61	<0.001
Scr (μmol/L)	79.89 ± 56.56	61.47 ± 44.71	0.001
UA (μmol/L)	315.74 ± 109.11	303.80 ± 99.96	0.292
TG (mmol/L)	1.44 ± 0.86	2.33 ± 2.29	<0.001
TC (mmol/L)	4.24 ± 1.07	4.48 ± 1.24	0.071
HDL-C (mmol/L)	0.99 ± 0.27	1.04 ± 0.28	0.101
LDL-C (mmol/L)	2.39 ± 0.71	2.55 ± 0.80	0.056

**Figure 1:**
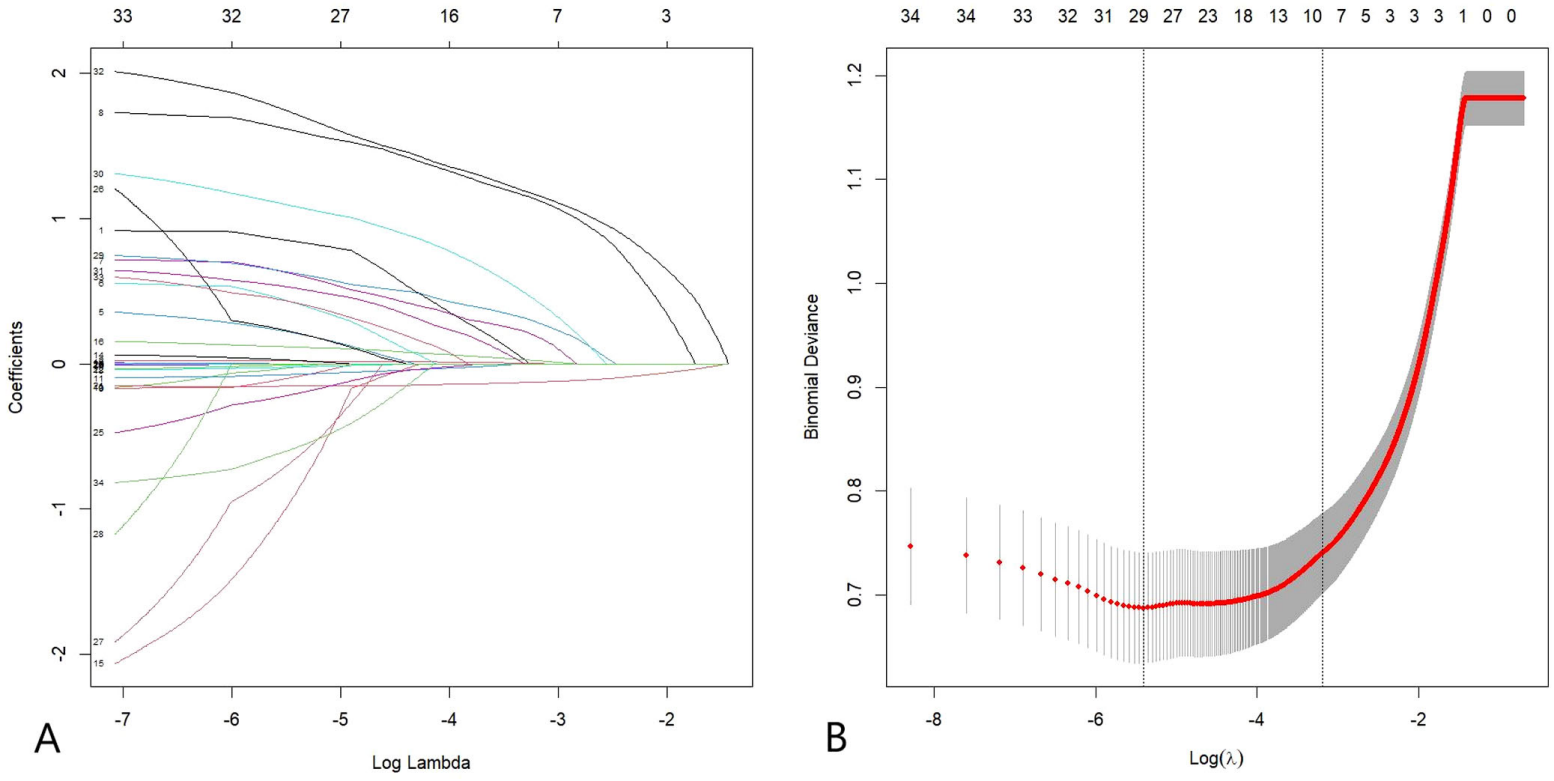
**(A)** Coefficient profiles of the 34 candidate variables. Each curve represents the trajectory of a coefficient as the value of the regularization parameter log(λ) changes. As λ increases, more coefficients shrink to zero, indicating variable selection. **(B)** Ten-fold cross-validation for tuning parameter selection. The plot shows binomial deviance against log(λ), with two vertical dashed lines indicating the optimal λ values: the left one corresponds to the minimum deviance, and the right one corresponds to the most regularized model within one standard error of the minimum (1-SE rule).

To identify risk factors for DFU, the occurrence of DFU in the modeling cohort was designated as the dependent variable, with all 34 candidate variables included as potential predictors. Given the potential multicollinearity among variables and to prevent overfitting, LASSO regression with tenfold cross-validation was employed for variable selection (**Figures 1A, B**, **Supplementary Table 1**). Based on the 1-SE rule, the optimal penalty parameter λ was determined as λ + 1 = 0.042. This yielded eight significant predictors: age, CI, ABI, WBC, ALB, UACR, family history of diabetes, and DPN.

### 3.3 Multivariate logistic regression analysis in the modeling cohort

The eight predictors identified by LASSO regression were included in the multivariate logistic regression model. The results indicated that older age (odds ratio [OR] = 1.032, 95% confidence interval [CI]: 1.005–1.062, *P* = 0.021), higher WBC (OR = 1.127, 95% CI: 1.006–1.270, *P* = 0.043), abnormal ABI (≥1.3 or ≤0.9) (OR = 5.447, 95% CI: 2.186–14.340, *P* < 0.001), UACR ≥30 mg/g (OR = 2.049, 95% CI: 1.062–3.936, *P* = 0.031), family history of diabetes (OR = 3.405, 95% CI: 1.666–7.039, *P* < 0.001), and DPN (OR = 5.084, 95% CI: 2.673–9.805, *P* < 0.001) emerged as significant independent risk factors for DFU in T2DM patients. Conversely, higher ALB (OR = 0.850, 95% CI: 0.786–0.915, *P* < 0.001) was identified as an independent protective factor. The final logistic regression equation was: Logit (P) = 0.758 + 0.032 × age + 0.119 × WBC + 1.695 × ABI + 0.718 × UACR + 1.225 × family history + 1.626 × neuropathy – 0.162 × ALB. In this model, CI (OR = 1.772, 95% CI: 0.762–4.119, *P* = 0.184) did not demonstrate a statistically significant association with DFU (**Table 3**).

**Table 3:** Influential factors for DFU identified by multivariate logistic regression analysis (modeling cohort).

Variable	β Coefficient	Standard Error	*P* value	OR	95% CI for OR
Constant	0.758	1.880	0.686	2.133	0.055–90.116
Age (years)	0.032	0.014	0.021	1.032	1.005–1.062
WBC (×10^9^/L)	0.119	0.059	0.043	1.127	1.006–1.270
ABI (≥1.3 or ≤0.9)	1.695	0.477	<0.001	5.447	2.186–14.340
UACR (≥30 mg/g)	0.718	0.333	0.031	2.049	1.062–3.936
Family history of diabetes	1.225	0.366	<0.001	3.405	1.666–7.039
DPN	1.626	0.331	<0.001	5.084	2.673–9.805
CI	0.572	0.430	0.184	1.772	0.762–4.119
ALB (g/L)	–0.162	0.039	<0.001	0.850	0.786–0.915

### 3.4 Development and validation of nomogram prediction model

**Figure 2:**
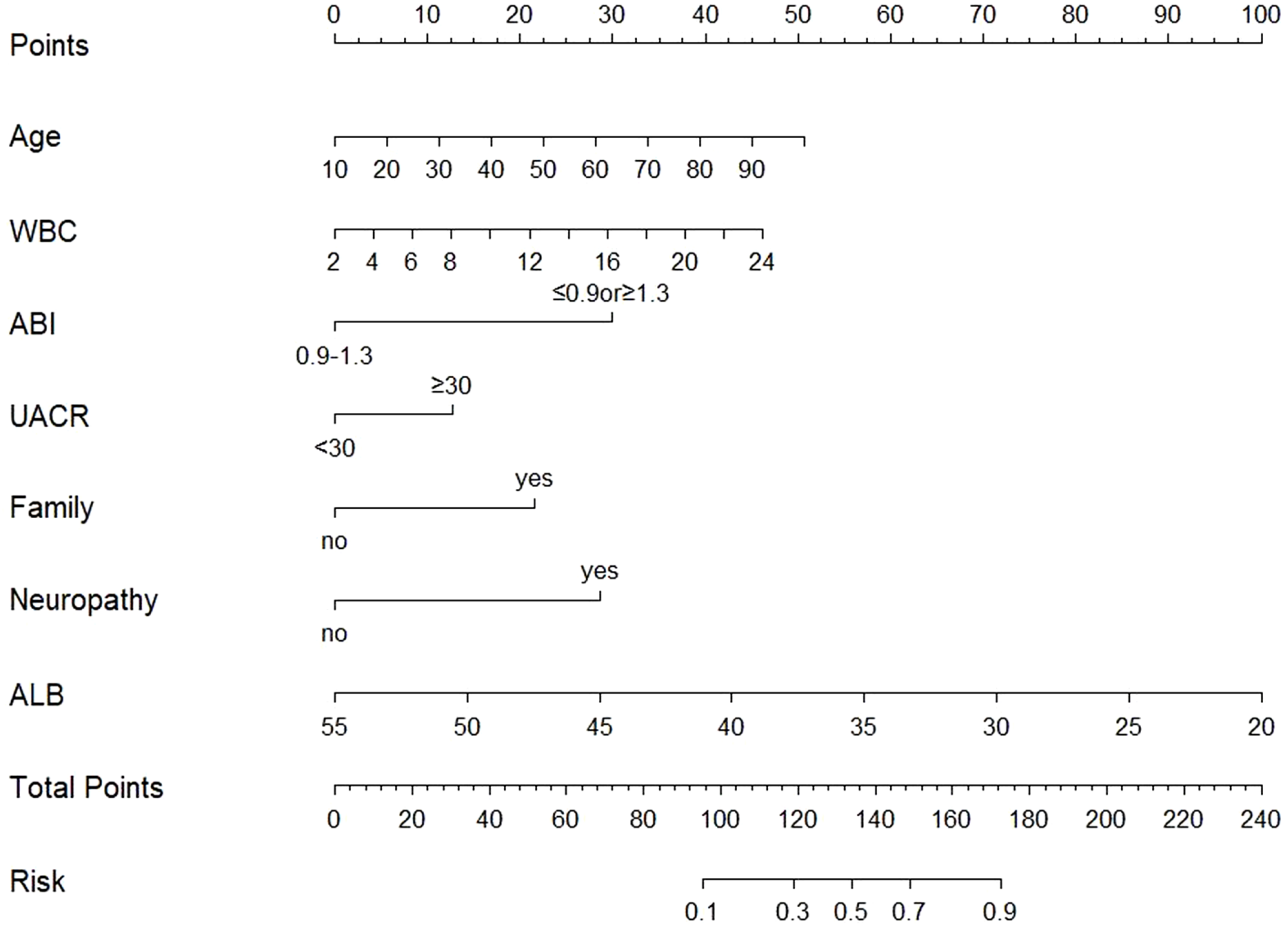
The nomogram integrates multiple predictors identified by multivariable logistic regression to estimate the probability of DFU. Each variable is assigned a point value according to its contribution to the model. The sum of the total points corresponds to the predicted risk at the bottom scale. Variable definitions: Age (years); WBC (white blood cell; ×10^9^/L); ABI (ankle–brachial index; ≤0.9 or ≥1.3 = abnormal, 0.9–1.3 = normal); UACR (urine albumin-to-creatinine ratio; ≥30 mg/g = abnormal, <30 mg/g = normal); Family (family history of diabetes; Yes = positive, No = negative); Neuropathy (diabetic peripheral neuropathy [DPN]; Yes = present, No = absent); ALB (albumin, g/L).

Derived from multivariate logistic regression analysis, the seven independent predictors of DFU in patients with T2DM (age, WBC, ABI, UACR, family history of diabetes, neuropathy, and ALB) were integrated into R version 4.1.3 to construct a nomogram for predicting DFU risk (**Figure 2**). In this nomogram, each variable was assigned points on the top scoring scale (ranging from 0 to 100). By summing the points for all variables, a total score is obtained, which corresponds to the probability of DFU on the bottom risk scale. A higher total score correlates with increased DFU risk in T2DM patients.

**Figure 3:**
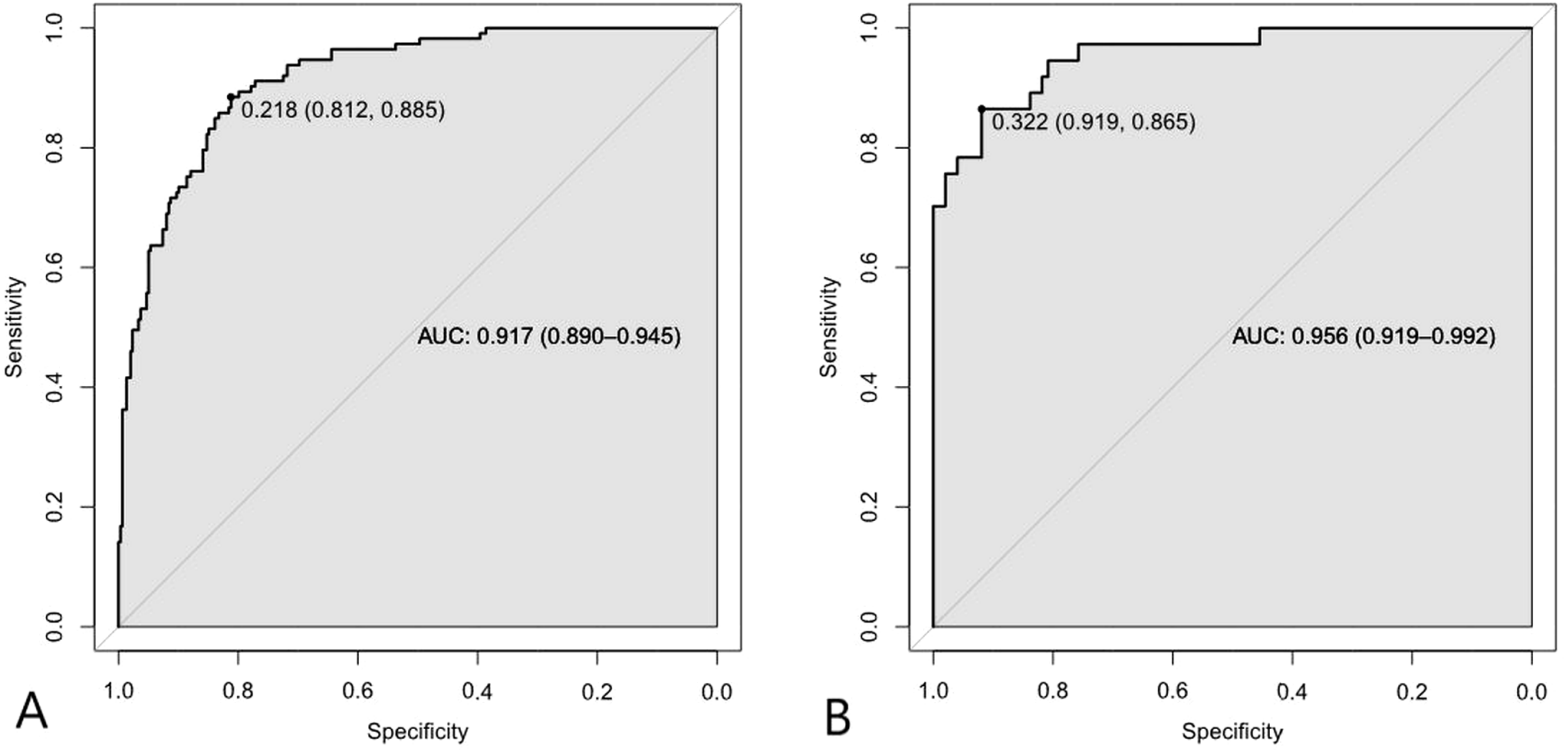
**(A)** ROC curve in the modeling cohort. The area under the curve (AUC) was 0.917 (95% CI: 0.890–0.945), indicating high discrimination. **(B)** ROC curve in the validation cohort. The AUC was 0.956 (95% CI: 0.919–0.992), demonstrating excellent external validity.

ROC curves affirmed the nomogram’s robust performance in both modeling and validation cohorts. In the modeling cohort (n=411), the AUC (95% CI) was 0.917 (0.890–0.945), with an optimal cut-off of 0.218 yielding sensitivity of 0.885 and specificity of 0.812 (**Figure 3A**). Similarly, in the validation cohort, the AUC was 0.956 (0.919–0.992), with a cut-off of 0.322 demonstrating sensitivity of 0.865 and specificity of 0.919 (**Figure 3B**). These AUC values confirm the nomogram’s excellent discriminative ability for identifying T2DM patients at risk of developing DFU.

**Figure 4:**
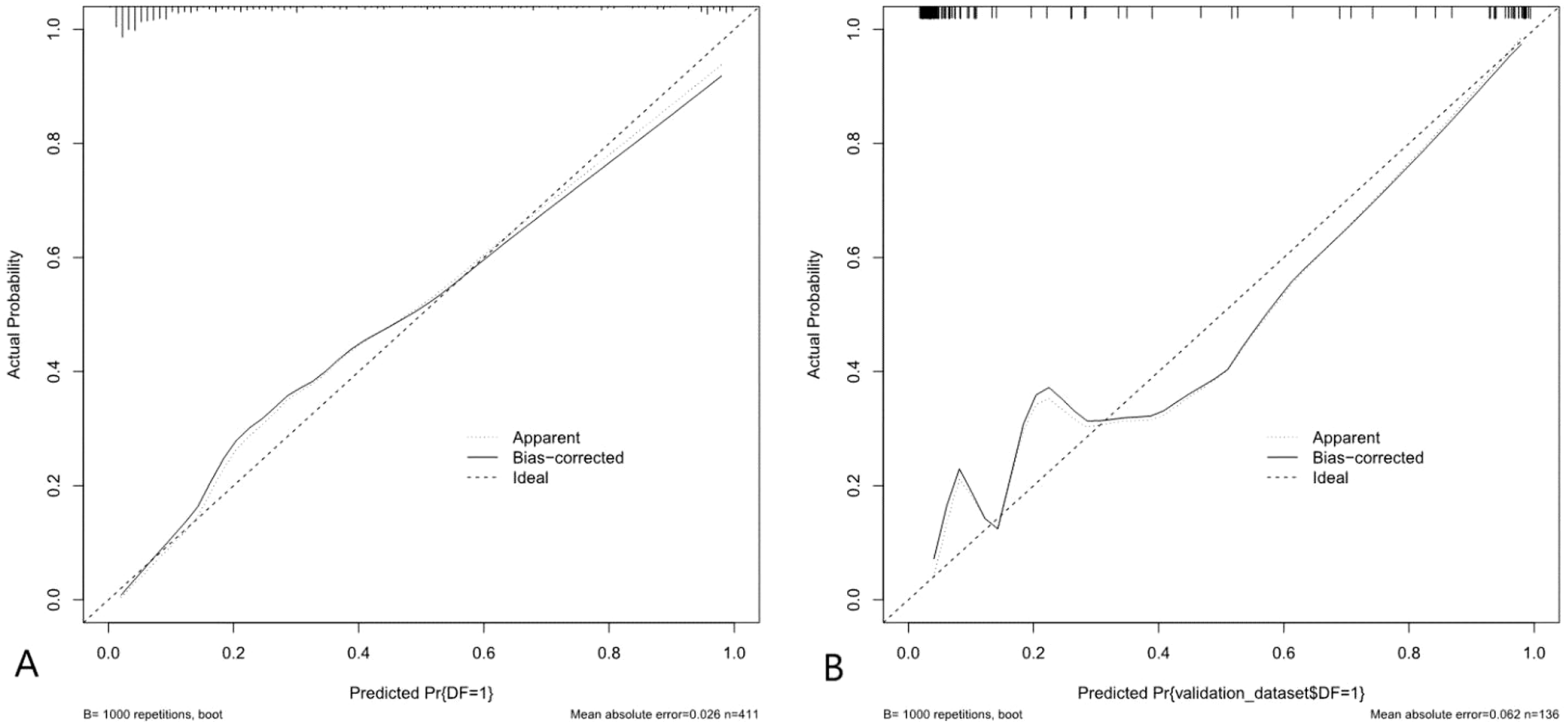
**(A)** Calibration in the modeling cohort. **(B)** Calibration in the validation cohort. The dashed line represents the ideal prediction; the solid line indicates the bias-corrected prediction via 1000 bootstrap samples. The proximity of the curves reflects good agreement between predicted and observed probabilities.

Bootstrap resampling with 1000 replicates for internal validation yielded a C-index of 0.917. The calibration curves presented in **Figure 4** demonstrate close alignment between predicted and observed DFU probabilities. This good agreement was statistically confirmed by the Hosmer-Lemeshow test, which showed no significant difference between the predicted and observed outcomes in either the modeling cohort (*P* = 0.649; **Figure 4A**) or the validation cohort (*P* = 0.345; **Figure 4B**).

**Figure 5:**
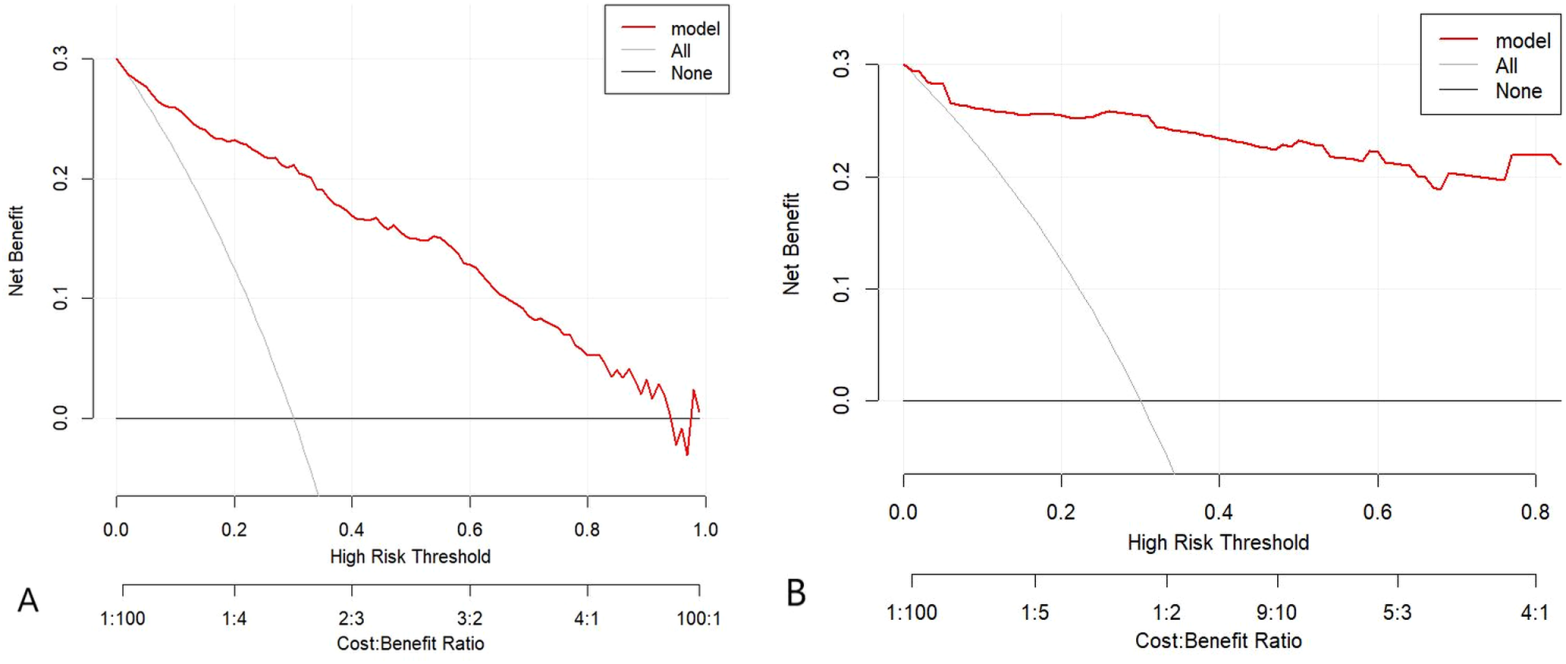
**(A)** DCA in the modeling cohort. **(B)** DCA in the validation cohort. The red line represents the net benefit of using the nomogram across a range of threshold probabilities, compared with “treat-all” (gray) and “treat-none” (black) strategies. The analysis demonstrates superior clinical utility when the threshold ranges from approximately 0.0 to 0.8.

DCA for the nomogram prediction model (**Figures 5A, B**) showed that across a threshold probability range of 0 to 0.8, the nomogram provided substantially higher net benefit for predicting the risk of DFU in T2DM patients compared to both treat-all and treat-none strategies. This wide applicability range attests to the nomogram’s reliability and clinical utility as a risk stratification tool in T2DM populations.

### 3.5 Visualization of nomogram prediction model

**Figure 6:**
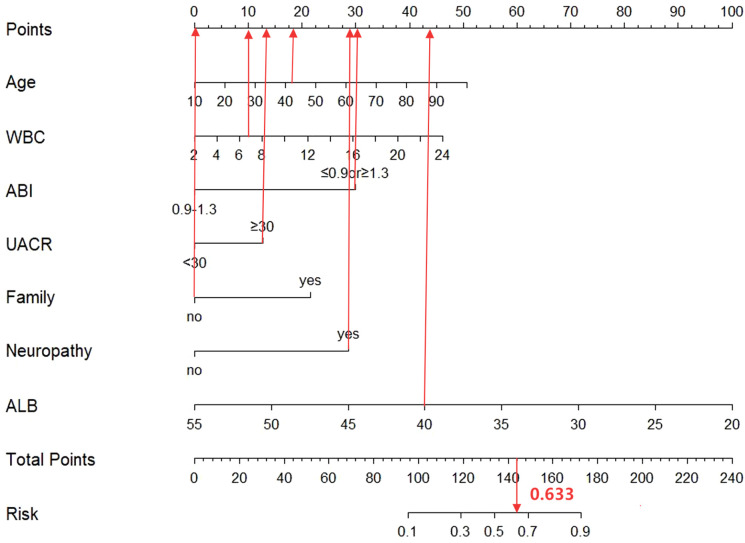
Visualization of the nomogram prediction model for the risk of DFU in T2DM Patients.

Consider a hypothetical 42-year-old male with T2DM exhibiting the following parameters: WBC 6.8×10^9^/L, ABI 0.7 (below 0.9), UACR 56 mg/g (above 30 mg/g), ALB 40 g/L, diagnosed with DPN, and no family history of diabetes. Based on the nomogram illustrated in **Figure 6**, this patient’s predicted probability of developing DFU is approximately 63.3%, surpassing the established risk threshold. Thus, according to DCA guidelines, this high-risk patient warrants immediate preventive interventions to mitigate DFU development risk.

## 4 Discussion

This study aimed to develop and validate a clinically applicable predictive model for identifying hospitalized patients with T2DM at high risk of developing DFU. By incorporating variables from five key domains—demographic and clinical characteristics, diabetic complications, physical examinations, laboratory parameters and treatment modalities—our analysis identified seven independent predictors: age, WBC, ABI, UACR, ALB, family history of diabetes, and DPN. These factors were incorporated into a nomogram, which was internally validated and demonstrated strong predictive performance. Notably, the model incorporates objective indicators-specifically ALB, ABI, and UACR-which are seldom combined in previous models, capturing nutritional status, vascular integrity, and renal microvascular injury, respectively. Feature selection using LASSO regression further enhanced the model’s accuracy and robustness. This nomogram facilitates personalized risk estimation in hospitalized T2DM patients, enabling clinicians to quantify the individual contribution of each predictor, identify and target modifiable risk factors, and implement tailored prevention strategies.

Advanced age was identified as a significant risk factor for DFU, in line with previous studies (14, 19, 21, 22). Aging is thought to increase DFU risk through cumulative plantar pressure, longer diabetes duration, poorer nutritional status, and a higher incidence of vascular complications, although some reports have also observed elevated risk in younger patients as well (23–25). It is worth noting that Crawford et al. reported that age was not predictive of DFU (12). Elevated WBC emerged as an independent predictor of DFU development, corroborating previous findings (14, 17, 26). This association likely reflects underlying chronic inflammation and microvascular dysfunction, both of which impair tissue repair and promote ulcer formation. Notably, a prospective cohort study demonstrated that for every 1,000 cells/μL increase in WBC, the adjusted risk of developing DFU increased by 30%, even after adjustment for glycemic control, DPN, and PAD (27).

Chronic complications such as DPN and PAD are significant contributors to the development of DFU and are well-recognized risk factors (18, 28–30). In this study, DPN and PAD were objectively assessed using nerve conduction velocity (NCV) and the ABI, respectively (31, 32). Abnormal ABI (OR = 5.447, 95% CI: 2.186–14.340) and DPN (OR = 5.084, 95% CI: 2.673–9.805) were identified as the top two independent predictors of DFU. DPN contributes to sensory loss and impaired pain perception, while PAD leads to ischemia and microvascular dysfunction, both of which impair wound healing and increase the risk of unnoticed injury and persistent ulceration. Additionally, UACR ≥30 mg/g was also identified as an independent risk factor, serving as an early marker of diabetic nephropathy (DN) and indicating systemic microvascular damage (33). DN frequently coexists with other microvascular complications, such as retinopathy and neuropathy, all of which are strongly associated with DFU onset (29, 34). Importantly, an elevated UACR ≥30 mg/g, even within the microalbuminuria range, reflects the coexistence of vascular dysfunction and neuropathy. These pathological processes collectively impair wound healing and compromise skin integrity, thereby increasing susceptibility to DFU. Thus, UACR may serve as a valuable biomarker for identifying high-risk individuals and supporting personalized prevention strategies. Our analysis also found that a family history of diabetes increased DFU risk, aligning with findings from Piran et al. and Tuglo et al. (26, 35). This association could be mediated by genetic susceptibility and suboptimal glycemic control. Lastly, reduced ALB, a marker of poor nutritional status, can also indirectly reflect the severity of microvascular damage. Hypoalbuminemia impairs collagen synthesis, increases infection risk, and hinders wound healing (13, 36). Collectively, these readily obtainable clinical variables facilitate early risk identification and enable personalized DFU prevention strategies.

Although the association between poor glycemic control and DFU is well documented, the relationship between specific glycemic indicators (e.g., HbA1c, FPG) and DFU risk remains inconsistent. A study report higher HbA1c levels among DFU patients, frequently in conjunction with socioeconomic disadvantages (37). However, in our analysis, neither HbA1c nor FPG achieved statistical significance in the multivariate model. Moreover, surprisingly, in univariate analysis the FPG levels of DFU patients were even lower than those of non-DFU patients. These findings are consistent with some studies (13, 24, 25, 38) but contradict others (39–41). One potential explanation for this discrepancy may be that all participants in our cohort were hospitalized; specifically, This selective admission may have minimized differences in admission glycemic levels and potentially inverted the expected trend. Additionally, DFU pathogenesis may depend more critically on the long-term effects of chronic hyperglycemia rather than point-in-time glycemic measurements at admission. Indeed, longer diabetes duration is generally associated with increased risk of developing DFU, particularly after exceeding 10 years (42). In this study, although diabetes duration was statistically significant in univariate analysis, it was not retained in the final LASSO regression model. This absence may be attributable to its effect largely being mediated indirectly through its strong link with end-organ complications, such as DPN and DN, reflected by UACR. These direct complications were prioritized as more potent predictors in the model.

Previous studies have reported a wide range of AUC values for DFU prediction models, ranging from 0.65 to 0.934. For example, Heald et al.’s logistic regression model in a primary care setting achieved an AUC of 0.65 (43), while Shao et al.’s nomogram model for elderly T2DM patients attained an AUC of 0.934 in external validation (19). Intermediate predictive performances have also been reported in prior studies, with AUC values of 0.81, 0.86, and 0.89 reported by Boyko et al. (11), Shi et al. (13), and Jiang et al. (14), respectively. These variations highlight that the predictive performance of models is substantially influenced by population characteristics, variable selection, and modeling approach. In this study, our nomogram demonstrated an AUC of 0.917 (95% CI: 0.890–0.945) in the modeling cohort and 0.956 (95% CI: 0.919–0.992) in the validation cohort, indicating excellent discriminative ability. As the field shifts toward personalized medicine for DFU prevention and management, this study tackles a key limitation of previous models, which often relied on discrete thresholds or risk-stratified scores for risk factors without quantifying their graded effects (14, 19). By integrating both categorical and continuous variables into a nomogram framework, our model provides a more precise, reliable, and individualized-and thus clinically applicable-risk prediction tool for DFU.

This study has certain limitations. First, the retrospective design and single-center nature, combined with a relatively small sample size, may introduce selection and information bias, thereby affecting the generalizability of the findings. The retrospective design was deliberately adopted to enable efficient model development based on real-world inpatient data, providing an empirical basis for future large-scale prospective studies. Although this approach facilitates the preliminary screening of potential predictors, it inherently constrains causal inference. Second, some key variables, such as education level, socioeconomic status, behavioral factors (e.g., footwear habits, treatment adherence), and prior foot complications (e.g., ulceration, amputation, deformities, and calluses), were not systematically recorded in electronic medical records. The absence of these variables limited the model’s comprehensive scope. Future studies should incorporate structured data collection to capture these psychosocial and behavioral dimensions. Third, the lack of time-to-event data precluded longitudinal risk prediction. Although internal validation using bootstrap resampling was performed, validation strategies were constrained by the limited sample size and event frequency. Future studies could incorporate k-fold cross-validation to further assess model stability. Crucially, the absence of external validation constitutes a major limitation. To address these issues, we have planned a prospective, multicenter validation study in collaboration with regional hospitals and primary care clinics. We will consecutively enroll hospitalized T2DM patients, prospectively collect predictor variables, and evaluate model discrimination and calibration over a 24-month follow-up period. This effort aims to assess model generalizability across care settings, refine risk thresholds, and support its clinical translation.

## 5 Conclusions

In conclusion, this study identified age, WBC, ABI, UACR, family history of diabetes, DPN, and ALB as independent predictors of DFU in patients with T2DM. Leveraging these predictors, a nomogram model was developed and internally validated to predict the risk of DFU. The model demonstrated favorable performance and high clinical utility, providing a valuable clinical tool for identifying high-risk T2DM patients, guiding early interventions, and ultimately reducing the incidence of DFU. To further evaluate its generalizability and facilitate clinical translation, a prospective multicenter validation study has been planned.

## Data Availability

The original contributions presented in the study are included in the article/**Supplementary Material**. Further inquiries can be directed to the corresponding author.
