# Supplementary material for: Development and validation of a nomogram for predicting diabetic foot ulcer risk in patients with type 2 diabetes mellitus
Source: Front Endocrinol (Lausanne). 2025 Jun 30;16:1555163. doi: 10.3389/fendo.2025.1555163 (PMC12256247; doi:10.3389/fendo.2025.1555163)
Supplement: Supplementary file 1 [file DataSheet1.pdf]

Supplementary Material

Supplementary Table 1. Variables selected by LASSO regression for predicting DFU (modeling cohort)

Variable	Coefficient (β)	Selected by LASSO
Gender	–	No
Hypertension	–	No
CAD	–	No
CI	0.175108107	Yes
ABI	1.119411155	Yes
Smoking	–	No
Alcoholism	–	No
UACR	0.276699783	Yes
Family history of diabetes	0.412623240	Yes
DR	–	No
DPN	1.152859477	Yes
OHA use	–	No
INS use	–	No

Age	0.001715801	Yes
BMI	–	No
Diabetes duration	–	No
FPG	–	No
FCP	–	No
HbA1c	–	No
Hb	–	No
RBC	–	No
WBC	0.020347627	Yes
PLT	–	No
ALT	–	No
AST	–	No
ALP	–	No
ALB	-0.123852992	Yes
BUN	–	No
Scr	–	No
UA	–	No

TG	–	No
TC	–	No
HDL-C	–	No
LDL-C	–	No
